# A Service Evaluation to Understand Factors Affecting Referrals to a Secondary Care Psychotherapy Department

**DOI:** 10.1192/bjo.2024.509

**Published:** 2024-08-01

**Authors:** Henrietta Rees

**Affiliations:** Surrey and Borders Partnership NHS Foundation Trust, Surrey, United Kingdom

## Abstract

**Aims:**

1. To evaluate sources and demographics of referrals to the Northwest Surrey Psychotherapy Service, a secondary care service covering Runnymede, Spelthorne, and Woking in Surrey, England. To compare these data with local population data to assess equality of access and whether any groups are underrepresented.
2. To evaluate what diagnoses are most frequent in those referred and the respective characteristics of those whose referrals are accepted for treatment and those who aren't.

**Methods:**

A retrospective case note review using SystmOne of all patients referred to Psychotherapy between January 2021 and June 2021. Data were extracted by hand: demographics (age, gender, ethnicity, sexual orientation, marital status, employment status, dependents, caring responsibilities, disability, religion), diagnosis, source and outcome of referral. Reasons for referrals not progressing were correlated with current service inclusion/exclusion criteria. Demographics were compared with local population data available from ONS and Surrey County Council.

**Results:**

Fifty-one people were referred, 10 (19.6%) males and 41(80.4%) females.

Twenty-six (51%) referrals were accepted. Amongst those referred, depression n = 15 (29%), post-traumatic stress disorder (PTSD) n = 15 (29%) and emotionally unstable personality disorder (EUPD) n = 13 (25%) were the most reported diagnoses. Persons with depression or EUPD were most frequently accepted for assessment and treatment. The most common reason for a referral not progressing was the patient experiencing active PTSD symptoms requiring prior stabilisation work n = 9 (17%) or the patient not opting in n = 5 (10%).

Referrals came from a range of sources, mostly general practitioners (GPs) n = 18 (35%) and MindMatters (primary care talking therapies) n = 8 (16%).

**Conclusion:**

Males were underrepresented in referrals to Psychotherapy and reasons may vary. It may be beneficial for referrers to be more proactive in considering and recommending referring males for psychotherapeutic input. Other groups were not significantly underrepresented compared with local population data, including ethnic minorities and those with protected characteristics.

Psychotherapy services frequently declined those suffering acute symptoms of PTSD; there may be a need to educate referrers that this is a likely exclusion criterion. Those who were declined on this basis were signposted to services offering stabilisation work, a positive finding in terms of our service facilitating access to ongoing care.

The sources of referrals suggest that GPs and MindMatters are important partners in identifying those needing psychotherapy services. Some referrals were inappropriate, and clearer referral criteria may be helpful. Some people declined assessments or treatment, which may indicate a need for more outreach or education on the potential benefits of psychotherapy services.

